# Authentication of the Geographical Origin of Margarines and Fat-Spread Products from Liquid Chromatographic UV-Absorption Fingerprints and Chemometrics

**DOI:** 10.3390/foods8110588

**Published:** 2019-11-19

**Authors:** Sanae Bikrani, Ana M. Jiménez-Carvelo, Mounir Nechar, M. Gracia Bagur-González, Badredine Souhail, Luis Cuadros-Rodríguez

**Affiliations:** 1 Department of Chemistry, Faculty of Sciences, University of Abdelmalek Essaâdi, Av. Sebta, Mhannech II, 93002 Tetouan, Morocco; sanae24bikrani@gmail.com (S.B.); mnechar@gmail.com (M.N.); bssouhail@yahoo.fr (B.S.); 2 Department of Analytical Chemistry, Faculty of Sciences, University of Granada, C/ Fuentenueva, s/n, E-18071 Granada, Spain; mgbagur@ugr.es (M.G.B.-G.); lcuadros@ugr.es (L.C.-R.)

**Keywords:** liquid chromatography fingerprinting, food authentication, margarines and spreads, multivariate classification

## Abstract

Fat-spread products are a stabilized emulsion of water and vegetable oils. The whole fat content can vary from 10 to 90% (*w*/*w*). There are different kinds, which are differently named, and their composition depends on the country in which they are produced or marketed. Thus, having analytical solutions to determine geographical origin is required. In this study, some multivariate classification methods are developed and optimised to differentiate fat-spread-related products from different geographical origins (Spain and Morocco), using as an analytical informative signal the instrumental fingerprints, acquired by liquid chromatography coupled with a diode array detector (HPLC-DAD) in both normal and reverse phase modes. No sample treatment was applied, and, prior to chromatographic analysis, only the samples were dissolved in n‑hexane. Soft independent modelling of class analogy (SIMCA) and partial least squares-discriminant analysis (PLS-DA) were used as classification methods. In addition, several classification strategies were applied, and performance of the classifications was evaluated applying proper classification metrics. Finally, 100% of samples were correctly classified applying PLS-DA with data collected in reverse phase.

## 1. Introduction

Margarine is a water-in-oil emulsion derived from vegetable and/or animal fats, with a fat content ranging from 80 to 90% (*w*/*w*) and a milk fat content of no more than 3% (*w*/*w*) [1,2]. This emulsion remains solid at 20 °C. It was discovered by Hippolyte Mège Mouriés, a French chemist, who patented his product in France and Britain in 1869. The product was described at that time as a blend of the glycerol esters of oleic and margaric acids and was therefore called oleo-margarine. Margaric acid was thought to be heptadecanoic acid (17:0), but currently, it is known that it was really a eutectic mixture of palmitic (16:0) and stearic (18:0) acids [3]. “Fat-spread” is the generic name that is applied to any emulsion with a fat content ranging from 10 to 90%, whereas the term “vegetable shortening” or just “shortening” refers to all fat produced from vegetal oils that are hydrogenated, i.e., they stay semisolid at room temperature. According to this, the fat content of shortening is 100%.

The European legislation currently in force distinguishes the following types, regarding fat content [4]: Margarine, three-quarter fat margarine, and half-fat margarine. The half-fat margarine is also named minarine or halvarine by Codex Alimentarius terminology [2]. On the other hand, Moroccan legislation is less restrictive and does not consider different kinds of margarine concerning fat content. In fact, margarine is defined as “any food fat substance, which is not butter or lard, but resembles butter and it is produced for using as butter” [5]. The milk fat content cannot exceed 10% (*w*/*w*). In order to simplify matters, and because it would not have major repercussions on the study, the term “margarine/spread” will be used in a broad sense in this paper, regardless of fat content.

For the manufacturing industrial process, the vegetal oils are heated at their melting temperature (approximately 40 °C) and mixed with the additives and emulsifiers to achieve a homogeneous mixture. In order to obtain a solid consistency of fat, the mixture is slowly cooled down and subjected to a hydrogenation process to produce fat saturation due to the unsaturated bonds of fatty acids of the vegetable oils breaking up [6].

The properties of margarines/spreads mainly depend on the characteristics of the vegetable oils, which are the major ingredients of the product, and the additives. The fat source is usually either soybean oil or sunflower oil blended with a hydrogenated vegetable oil, typically in the ratio 3:1 [7]. Other commodity vegetable oils include rape/canola, cottonseed, palm, palm kernel, and coconut, which may have been fractionated, blended, hydrogenated in varying degrees, and/or interesterified. Fish oil (hydrogenated or not) may also be included. Recent trends in the margarine market also consider mixtures with "healthy" vegetable oils with a low content of trans-saturated acid (e.g., high oleic sunflower or olive oils), as well as the addition of sterols. Regarding additives, these include surface-active agents, proteins, salt, and water, along with preservatives, flavours, and vitamins [8].

From the chemical point of view, the fat fraction of margarines/spreads is mainly composed of triacylglycerols (TAGs) [9]. Some data on the TAG composition of margarine/spread have been reported [10], but, as far as the authors are concerned, only one paper has been published, devoted to this matter [11]. The main compositional parameter being important for margarine/spread quality and healthy features is focused on the fatty acid (FA) profile [12,13,14]. The FA profile is commonly analysed using gas chromatography after derivatisation of them in the corresponding methyl esters. This approach provides incomplete chemical information and does not allow the knowledge of the variability linked to the combination of three FAs in the TAGs.

Conventional chromatographic techniques have been used successfully for the qualitative and quantitative determination of TAGs [15,16], mainly high-performance liquid chromatography (HPLC) in both normal and reverse phase modes [17,18,19]. The coupling of mass spectrometry to chromatographic instruments has drastically increased the analytical capabilities [20,21], and the regioisomeric and enantiomeric analysis of triacylglycerols is already affordable [22], but the chromatographic signals obtained from TAG profiling methods are never specific enough due to the great variety of isomers present in low proportion. In addition, 1H NMR has also been proven to be very useful in determining the composition in acyl groups of margarine samples, in only a few minutes and with minimal or no sample pretreatment [1,23]. Some examples of TAG profiling in some closely related margarine/spread products as milk and dairy foods can be found in references [24,25].

Nevertheless, the TAGs profile is characteristic of each vegetable oil, according to its botanical species, genus, or variety, which has a characteristic lipid profile. Consequently, TAGs profiling is both a useful and reliable tool in identifying vegetable oils and/or fraud detection [16] in order to verify the stated composition of margarines/spreads and authenticate them. Moreover, to our knowledge, there are no antecedents describing the comparison and classification of different margarines/spreads according to their geographical origin.

Another relatively alternative way to identify each vegetable oil using TAG analytical information is applying the fingerprinting methodology. This applies nonspecific instrumental signals where all the implicit, but nonevident, information contained in the analytical signal acquired from the samples is used, not being necessary to profile each chemical species present in the working solution. In this sense, signals coming directly from the measurement device or detector coupled with the chromatographic instrument are treated as a whole, and by means of advanced chemometric tools, as multivariate data analysis to extract, it is possible to reduce and process the extensive datasets in order to build proper multivariate models for classification or quantification purposes [26,27]. This methodology has become one of the most efficient and comprehensive methods to verify food identity [28].

This paper presents a multivariate qualitative analytical method for authenticating the geographical origin of margarines and fat-spread products. Several multivariate chemometrics tools were applied, such as principal components analysis (PCA), soft independent modelling by class analogy (SIMCA), and partial least squares-discriminant analysis (PLS-DA). As an analytical information source for building the multivariate models, both normal and reverse phase liquid chromatographic fingerprints were used. For this purpose, the analytical signals were acquired using a diode array detector (DAD) coupled with a high-performance liquid chromatographic (HPLC) system. Three strategies were tested to build the classification models: Two input-class, pseudo two input-class, and one input-class classification. The results from each classification method and strategy were compared and ranked on the basis of several classification performance metrics.

## 2. Materials and Methods

### 2.1. Chemicals and Samples

All solvents employed were HPLC-grade. Isopropanol and n-hexane were purchased from PANREAC Química (Barcelona, Spain), and acetonitrile was provided by VWR International Eurolab, S.L. (Barcelona, Spain).

A total of 35 margarine samples of different trade names or brands were analysed: 17 from Spain, 1 from France, 1 from Belgium, 1 from Germany, 1 from the Netherlands, 1 from the United Kingdom, and 13 from Morocco. Table 1 shows a description of the kind of vegetable oil employed in the manufacture of the products.

### 2.2. Sample Preparation

10% (*w*/*w*) solutions of margarine in n-hexane were prepared. The solutions were stirred for 5 min, then they were decanted and the supernatant was passed through a polytetrafluoroethylene (PTFE) membrane syringe filter (0.22 µm), and the resultant solutions were stored at −20 °C until analysis. Before the chromatographic analysis, the solutions were again diluted with n-hexane at a 1:1 ratio.

### 2.3. Instrumentation/Chromatography Conditions

Analysis using normal and reverse liquid chromatography coupled with a diode-array detector, (NP)HPLC‑DAD and (RP)HPLC‑DAD, respectively, was carried out with an Agilent 1260 series liquid chromatograph (Santa Clara, CA, USA), equipped with a column thermostat (Eppendorf CH30), a quaternary pump, and degasser auto sampler. Agilent ChemStation OpenLab CDS software (rev. C.01.09) for LC systems was used to collect and record data.

(NP)HPLC‑DAD analysis was carried out using a column Lichrospher 100 CN (length 25 cm × i.d. 4 mm, particle size 4 µm) provided by Merck (Darmstadt, Germany). The column temperature was constant at 30 °C and the mobile phase was composed of n/hexane/isopropanol (96:4, *v*/*v*) at a flow rate of 1.2 mL min^−1^. The run time was 29 min.

(RP)HPLC-DAD analysis was performed using the column DevelosilTM C30-UG-5 (length 25 cm × i.d. 4.6 mm, particle size 5 µm) from Nomura Chemical Co. (San Diego, CA, USA). During the analysis, the column temperature was at 50 °C. A mixture of acetronitre/isopropanol (40:60, *v*/*v*) was used as mobile phase at a flow rate of 1.2 mL min^−1^. The chromatographic run time was 30 min.

**Figure 1:**
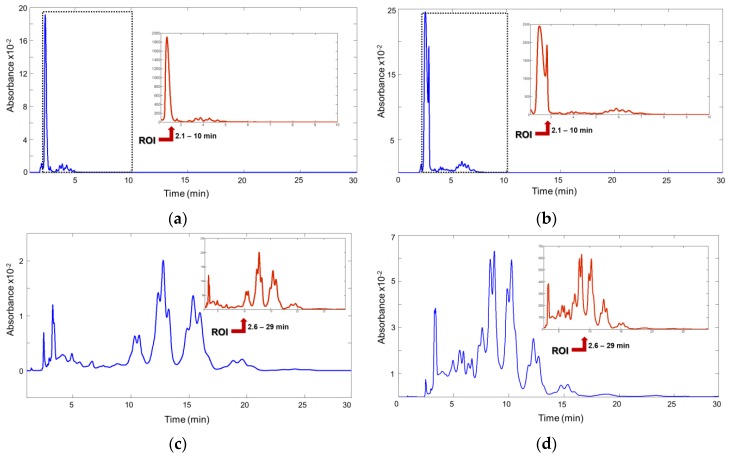
Chromatogram of a margarine/spread samples showing the region of interest used to build the classification models: (**a**,**b**) Normal phase from Morocco and Spain, respectively; (**c**,**d**) reverse phase from Morocco and Spain, respectively.

The injection volume was 20 µL, and the DAD spectra were acquired at 210 and 254 nm. Figure 1a shows the fingerprint of a sample from Morocco recorded at 210 nm, and Figure 1b shows a sample from Spain that recorded the same wavelength obtained using (NP)HPLC‑DAD. Likewise, Figure 1c,d displays the fingerprints of the same samples recorded at 210 nm using (RP)HPLC‑DAD, respectively.

With respect to the TAG composition of the blended fat, the obtained fingerprint depends on the proportion of unsaturated/saturated FAs. Note that only the unsaturated FAs generate a measurable signal as the saturated FAs are almost transparent to the UV-absorption detector at the working concentration. Consequently, fingerprints show specificity from the distribution of the unsaturated FAs into the different TAGs. This characteristic causes the UV-absorption fingerprints to be different to the fingerprint acquired from other more universal detectors used in edible fat analyses, such as refractive index (RID), evaporative light scattering (ELSD), and corona charged aerosol (CAD).

### 2.4. Chemometrics

The raw data files from each chromatogram were exported in “comma separated value” (CSV) format and then turned to “xls” format (Microsoft Excel). For (NP)HPLC-DAD and (RP)HPLC-DAD analysis, a data vector composed of 4500 and 4350 variables (each one a specific absorbance to a scanned wavelength) was collected for each sample, respectively. The chromatographic fingerprints were reproducible from sample to sample, and thus, no alignment process was applied. The only preprocessing data carried out was the mean centring of the dataset before the development of the models. PCA, SIMCA, and partial least squares-discriminant analysis (PLS-DA) methods were built using The Unscrambler software ver. 9.7 (CAMO, Oslo, Norway).

Three classification strategies were applied: one input-class (1iC), two input-class (2iC), and pseudo two input-class (*p*2iC) to develop the different Spain/Morocco binary classification methods. Two input-class is the conventional binary classification methodology in which the model is trained using samples from target and nontarget class. One input-class strategy is used only in class modelling methods such as SIMCA. In these, the model is trained only with the target class. This methodology presents an advantage in food authentication as it is only necessary to analyse samples from target class (genuine class). Nevertheless, the modelling methods are less reliable than discriminant analysis methods; thus, the pseudo two input-class is applied in order to employ discriminant methods when it is only possible to have samples from the “genuine class”. A more detailed description about these strategies is shown in the paper published by Jiménez-Carvelo et al. [29]. For each strategy, the original data set was randomly split into different sets: Training and validation set. For 2iC, the training set was made up of 20 margarine samples, and the external validation set was composed of the remaining margarine samples. For *p*2iC, the training set was made up of 10 samples and 6 solvent analytical blank replicates, and the validation set was composed of 20 samples. For 1iC, the training set was composed of 10 samples, and the validation set included 20 samples. Table 2 details the sample distribution regarding the geographical origin for each classification strategy.

Once all the classification models were validated, they were critiqued using the samples from the validation set, and new classification models were built. To follow, these final models were used to predict the continent origin of the margarine/spread samples from the European countries other than Spain (France, Belgium, Germany, the Netherlands, and the United Kingdom). These samples constitute the prediction set.

## 3. Results and Discussion

**Figure 2:**
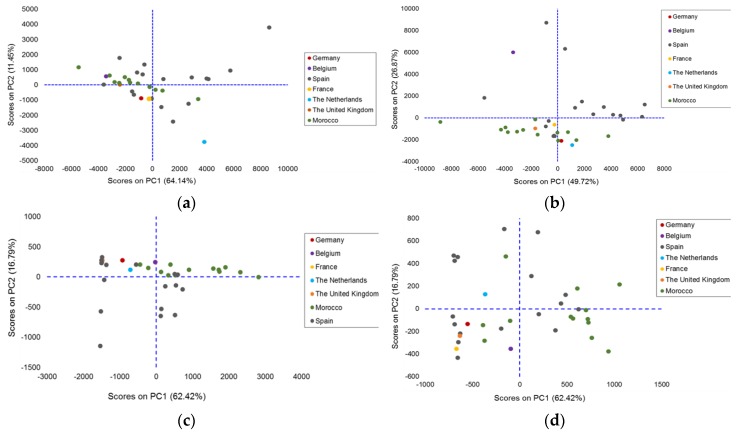
PCA scores obtained from the fingerprint data of the 35 margarine samples: PC1–PC2 plane of the chromatogram acquired at 210 nm: (**a**) In reverse phase; (**b**) In normal phase; and acquired at 254 nm: (**c**) In reverse phase; (**d**) In normal phase.

Firstly, four PCA models were built using the dataset composed of the whole fingerprint from each sample in both normal and reverse phase modes. Table 3 shows the number of PCs chosen for each model. Figure 2 shows the scores on the PC1–PC2 plane of the fingerprints acquired at 210 nm and 254 nm. The best groupings were found in the PCA models from data collected at 210 nm.

Once PCA models were evaluated, the three strategies (2iC, 1iC, and *p*2iC) were applied to develop the SIMCA and PLS-DA classification models. Nevertheless, the best results were found using the 2iC strategy from the 210-collecting dataset for both normal and reverse phases. Thus, the results shown in the following sections correspond with these datasets, considering the “Spanish class” as the target class in every case. It is important to emphasise that only the values of the performance metrics related to the target class provide useful information when the classification is used as a screening method, because the errors of the samples belonging to the nontarget class are not critical information as they all may be subjected to the confirmatory method.

### 3.1. SIMCA Methods

The application of SIMCA involves building a classification method in which each class of the training set is modelled independently (Spain model and Morocco model). Once the individual PC models were built, these were assembled to perform the classification of the samples. Two SIMCA models, for both the normal phase and reverse phase dataset, were then developed choosing four principal components (PCs).

**Figure 3:**
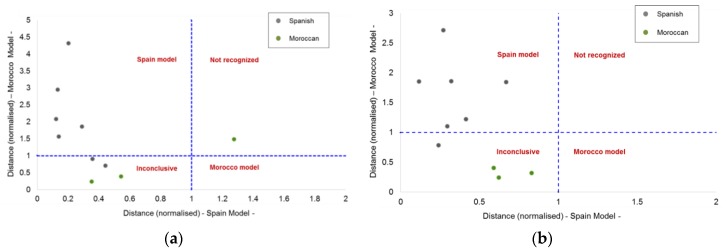
Coomans’ plot from (**a**) normal and (**b**) reverse liquid chromatography coupled with diode-array detector ((NP)HPLC-DAD and (RP)HPLC-DAD, respectively) datasets at 210 nm.

The classification results were evaluated, attending to Coomans’ plot. Figure 3 displays the Coomans’ plot of the two SIMCA models. It can be observed that some samples are located in the bottom-left quadrant; thus, these samples were considered inconclusive and they were not taken into account for estimation of the quality metrics for each model. Table 4 shows the results of the success/error contingencies, and Table 5 collects the quality metrics calculated for each model.

Coomans’ plot is a tool to graphically visualize principal groupings results in pairwise plots easily, in which the two axes represent the normalised orthogonal distances of all the samples with respect to each individual model. Ideally, the validation samples should be classified in one class or another (target or nontarget class). In real conditions, some validation samples could be assigned to both classes simultaneously, as these samples are considered inconclusive ones, or to neither of them (the samples are not recognized as belonging to any class).

### 3.2. PLS-DA Methods

The discriminant methods are generated through establishing the boundaries for the different categories defined by the training objects. PLS-DA is a latent variable-based method whose development involves two stages: (I) Firstly, a PLS regression model is established from the latent variables (LV) to establish limits between the classes, and then, (ii) a discriminant analysis (DA) is performed to classify the samples into a specific class.

Two PLS-DA models were built using five LVs from both normal and reverse liquid chromatography datasets. The “Spanish class” was defined by values equal to 0, while the “Moroccan class” was defined by a value of 1. The decision criterion established for the classification of the samples was a threshold value of 0.5, i.e., all the margarine samples with scores greater than 0.5 were classified to the Moroccan class, and margarine samples with scores lower than 0.5 were assigned to the Spanish class. In addition, for the purpose of improving the reliability of the validation and prediction results, an uncertainty interval was established as plus/minus 0.1 the settled threshold value for the training samples.

**Figure 4:**
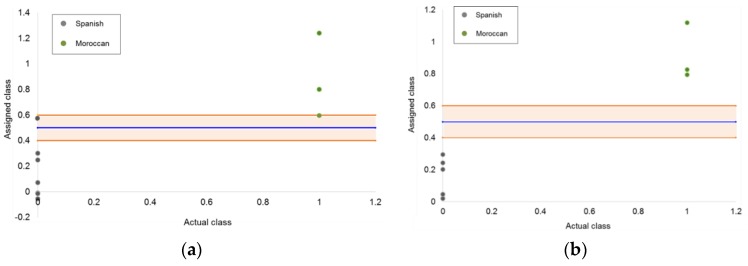
Classification plot of the partial least squares-discriminant analysis (PLS-DA) models from liquid chromatography (LC) data obtained in (**a**) normal phase and (**b**) reverse phase at 210 nm. The orange area represents the inconclusive area.

Figure 4 shows the classification plots obtained from PLS-DA methods. The blue line displays the classification threshold, and the orange strip represents the uncertainty region, within which any sample is stated as inconclusive.

The PLS-DA classification performances were evaluated, calculating the same quality metrics as SIMCA. These were estimated using the success/error contingency for each class in which samples of the validation set were arranged. Both contingency tables and quality metrics for the two PLS-DA classifiers established are shown in Table 6 and Table 7, respectively.

As can be seen, PLS-DA models provided better classification results than SIMCA ones. In particular, the model developed using the (RP)HPLC-DAD dataset was the best, as all the quality metrics were equal to 1.00 and there were not any samples classified as inconclusive. All the margarine/spread samples from the target class were well classified (probability = 0), and the samples from the nontarget class were also classified correctly (probability = 1).

**Figure 5:**
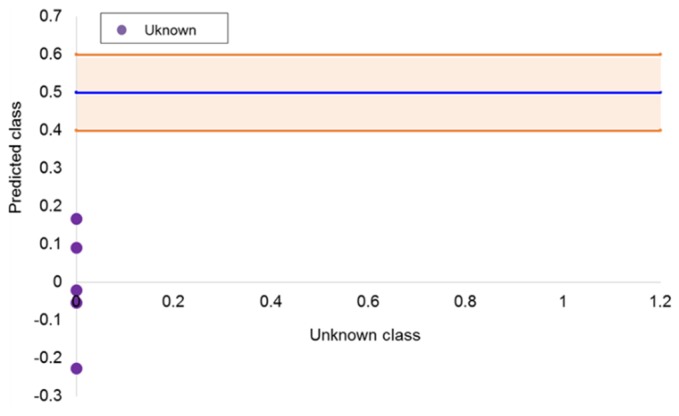
Class predictions plot for the samples from the European countries other than Spain.

As stated in Section 2.4, once SIMCA and PLS-DA models were correctly validated, the best model was applied to predict the class similarity of the margarine/spread samples from Belgium, France, Germany, the Netherlands, and the United Kingdom. For this purpose, the PLS-DA model built from the (RP)HPLC-DAD dataset was employed. The main goal of performing this classification was to test: (i) If the model was able to classify these samples as inconclusive as they belonged to neither Spanish nor Moroccan classes, and (ii) if the model was able to find similarities with any of the modelling classes (originating from Spain or Morocco). As can be seen in Figure 5, the model classified the five samples in the Spanish class. Probably, the overriding reason is related to the manufacturing process, because in Europe, there is an extensive and descriptive legislation that the margarine/spread products should be similar enough and all alternative to those manufactured in Morocco.

## 4. Conclusions

A methodology for the discrimination of margarine and fat-spread foodstuffs from Spain and Morocco using the fingerprinting methodology was described. Normal and reverse phase liquid chromatography coupled with UV-absorption was used as an analytical technique to acquire the 210 and 254 nm chromatographic fingerprints. In addition, different multivariate classification methods and strategies were tested, and the 210 nm PLS-DA models were found to perform better than the SIMCA ones, as all the samples were correctly classified.

It has to be stressed that the proposed methodology could also be applied to differentiate margarine and fat-spread products produced in any European country from the ones manufactured in Morocco, because all the Europe samples gave similar results of belonging to the Spanish class. Even so, further studies are necessary to test this hypothesis, which is currently being performed by the authors.

## Figures and Tables

**Table 1:** Types of vegetable oils present in the samples.

Sample No.	Origin	Vegetable Oil
1	Spain	Sunflower, palm, and corn
2		Sunflower and palm
3		Sunflower and palm
4		Sunflower, coconut, and canola
5		Sunflower, linseed, coconut, and canola
6		Sunflower, linseed, and palm
7		Sunflower, linseed, palm, and canola
8		Olive, sunflower, linseed, and palm
9		Sunflower, linseed, and palm
10		Sunflower, linseed, and palm
11		Olive, sunflower, linseed, and palm
12		Soybean, sunflower, linseed, and palm
13		Sunflower and palm
14		Olive, sunflower, linseed, coconut, and shea
15		Sunflower, linseed, and palm
16		Sunflower, coconut, canola, and shea
17		Soybean, sunflower, linseed, and palm
18	Morocco	-
19		-
20		Soybean
21		-
22		-
23		Soybean and corn
24		Soybean
25		-
26		Corn
27		Soybean
28		Soybean
29		Soybean
30		Soybean
31	The Netherlands	Sunflower, palm, and coconut
32	United Kingdom	Sunflower, palm, and rapeseed
33	France	Rapeseed, palm, olive, and sunflower
34	Germany	Soybean, palm, rapeseed, and coconut
35	Belgium	Palm, coconut, and canola

The hyphen “-” signifies that the kind of vegetable oil present in the samples is unknown.

**Table 2:** Distribution of the samples used in the different classification datasets regarding geographical origin.

		SIMCA		PLS-DA	
Dataset	Origin	(1ic)	(2ic)	(*p*2ic)	(2ic)
Training set	Spain	10	10	10	10
	Morocco	--	10	--	10
	Blank	--	--	6	--
Validation set	Spain	7	7	7	7
	Morocco	13	3	13	3
Prediction set	Europe (other than Spain)	--	--	--	5

**Table 3:** Characteristics of the principal components analysis (PCA) models.

	(RP)HPLC-DAD		(NP)HPLC-DAD	
Wavelength	PCs	% Var	PCs	% Var
210 nm	4	90.00	4	95.00
254 nm	4	99.00	4	93.00

**Table 4:** Soft independent modelling of class analogy (SIMCA) classification results for the validation set.

**(NP)HPLC-DAD**						
			7	3	**10**	
	**Assignation**	**O**	0	1	1	
		**I**	2	2	4	
		**nT**	0	0	0	
		**T**	5	0	5	
			**T**	**nT**		
			**Actual**			
**(RP)HPLC-DAD**						
			7	3	**10**	
	**Assignation**	**O**	0	0	0	
		**I**	1	3	4	
		**nT**	0	0	0	
		**T**	6	0	6	
			**T**	**nT**		
			**Actual**			

T: Target class (Spanish class); nT: Nontarget class (Moroccan class); I: Inconclusive samples; O: Samples not considered as belonging to any class.

**Table 5:** Quality metrics of the SIMCA models.

Parameter	(NP)HPLC-DAD	(RP)HPLC-DAD
Sensitivity (or Recall)	0.71	0.86
Specificity	0.00	0.00
Positive predictive value (Precision)	1.00	1.00
Negative predictive value	--	--
Youden index	−0.29	−0.14
Positive likelihood rate	0.71	0.86
Negative likelihood rate	--	--
*F*-measure	0.83	0.92
Discriminant power	--	--
Efficiency (or Accuracy)	0.50	0.60
AUC (Correctly classified rate)	0.36	0.43
Matthews correlation coefficient	--	--
Kappa coefficient	0.23	0.31

The hyphen “-” signifies that the performance feature cannot be determined.

**Table 6:** PLS-DA classification results for the validation set.

**(NP)HPLC-DAD**						
			7	3	**10**	
	**Assignation**	**O**	0	0	0	
		**I**	1	1	2	
		**nT**	0	2	2	
		**T**	6	0	6	
			**T**	**nT**		
			**Actual**			
**(RP)HPLC-DAD**						
			7	3	**10**	
	**Assignation**	**O**	0	0	0	
		**I**	0	0	0	
		**nT**	0	3	3	
		**T**	7	0	7	
			**T**	**nT**		
			**Actual**			

T: Target class (Spanish class); nT: Nontarget class (Moroccan class); I: Inconclusive samples; O: Samples not considered as belonging to any class.

**Table 7:** Quality metrics of the PLS-DA models.

Parameter	(NP)HPLC-DAD	(RP)HPLC-DAD
Sensitivity (or Recall)	0.86	1.00
Specificity	0.67	1.00
Positive predictive value (Precision)	1.00	1.00
Negative predictive value	1.00	1.00
Youden index	0.52	1.00
Positive likelihood rate	2.57	--
Negative likelihood rate	0.21	0.00
*F*-measure	0.92	1.00
Discriminant power	0.60	--
Efficiency (or Accuracy)	0.80	1.00
AUC (Correctly classified rate)	0.76	1.00
Matthews correlation coefficient	0.76	1.00
Kappa coefficient	0.62	1.00

The hyphen “-” is signifying that the performance feature cannot be determined.

## References

[1] Guillén M.D., Ibargoitia M.L., Sopelana P., Caballero B., Finglas P.M., Toldrá F. (2016). Margarine: Composition and analysis. Encyclopedia of Food and Health.

[2] (2017). Codex Stan 256-2007 Standard for Fat Spreads and Blended Spreads.

[3] Gunstone F.D. (2008). Oils and Fats in the Food Industry.

[4] (2017). Regulation (EU) No 1308/2013 Establishing a Common Organization of the Markets in Agricultural Products.

[5] (1970). Décret nº 1153-55 du 18 kaada 1389 (26 Janvier 1970) Portant Réglementation pour L’application du Dahir du 23 Kaada 1332 (14 Octobre 1914) sur la Répression des Fraudes en ce qui Concerne la Fabrication et la Vente de la Margarine, B.O nº 2988 du 04/02/1970.

[6] Wassell P., Rajah K.K. (2014). Bakery fats. Fats in Food Technology.

[7] Keogh M.K., Fox P.F., McSweeney P.L.H. (2006). Chemistry and technology of butter and milk fat spreads. Advanced Dairy Chemistry: Lipids.

[8] Gunstone F.D. (2004). The Chemistry of Oils and Fats: Sources, Composition, Properties and Uses.

[9] Marini F., Caballero B., Finglas P.M., Toldrá F. (2016). Triacylglycerols: Characterization and determination. Encyclopedia of Food and Health.

[10] List G.R., Steidley K.R., Neff W.E. (2000). Commercial spreads formulation, structures and properties. Inform.

[11] Craig Byrdwell W., Neff W.E., List G.R. (2001). Triacylglycerol analysis of potential margarine base stocks by high-performance liquid chromatography with atmospheric pressure chemical ionization mass spectrometry and flame ionization detection. J. Agric. Food Chem..

[12] Larqué E., Garaulet M., Pérez Llamas F., Zamora S., Tebar F.J. (2003). Fatty acid composition and nutritional relevance of most widely consumed margarines in Spain. Grasas y Aceites.

[13] Kroustallaki P., Tsimpinos G., Vardavas C.I., Kafatos A. (2011). Fatty acid composition of Greek Margarines and their change in fatty acid content over the past decades. Int. J. Food Sci. Nutr..

[14] Garsetti M., Balentineb D.A., Zock P.L., Blom W.A.M., Wanders A.J. (2016). Fat composition of vegetable oil spreads and margarines in the USA in 2013: A national marketplace analysis. Int. J. Food Sci. Nutr..

[15] Buchgraber M., Ulberth F., Emons H., Anklam E. (2004). Triacylglycerol profiling by using chromatographic techniques. Eur. J. Lipid Sci. Technol..

[16] Indelicato S., Bongiornob D., Pitonzo R., Di Stefano V., Calabrese V., Indelicato S., Avellone G. (2017). Triacylglycerols in edible oils: Determination, characterization, quantitation, chemometric approach and evaluation of adulterations. J. Chromatogr. A.

[17] Lısa M., Lynen F., Holcapek M., Sandra P. (2007). Quantitation of triacylglycerols from plant oils using charged aerosol detection with gradient compensation. J. Chromatogr. A.

[18] Rombaut R., De Clercq N., Foubert I., Dewettinck K. (2009). Triacylglycerol analysis of fats and oils by evaporative light scattering detection. J. Am. Oil Chem. Soc..

[19] de la Mata Espinosa P., Bosque Sendra J.M., Cuadros Rodríguez L. (2011). Quantification of triacylglycerols in olive oils using HPLC-CAD. Food Anal. Methods.

[20] Laakso P. (2002). Mass spectrometry of triacylglycerols. Eur. J. Lipid Sci. Technol..

[21] Xu S.-L., Wei F., Xie Y., Xin L., Dong X., Chen H. (2018). Research advances based on mass spectrometry for profiling of triacylglycerols in oils and fats and their applications. Electrophoresis.

[22] Rezanka T., Pádrová K., Sigler K. (2017). Regioisomeric and enantiomeric analysis of triacylglycerols. Anal. Biochem..

[23] Sopelana P., Arizabaleta I., Ibargoitia M.L., Guillén M.D. (2013). Characterisation of the lipidic components of margarines by 1H nuclear magnetic resonance. Food Chem..

[24] Fontecha J., Juárez M., de la Fuente M.A., Nollet L.M.L., Toldra F. (2010). Triacylglycerols in dairy foods. Handbook of Dairy Foods Analysis.

[25] Omar K.A., Gounga M.E., Liu R., Mwinyi W., Aboshora W., Ramadhan A.H., Sheha K.A., Wang X. (2017). Triacylglycerol composition, melting and crystallization profiles of lipase catalysed anhydrous milk fats hydrolysed. Int. J. Food Prop..

[26] Bosque Sendra J.M., Cuadros Rodríguez L., Ruiz Samblás C., de la Mata A.P. (2012). Combining chromatography and chemometrics for the characterization and authentication of fats and oils from triacylglycerol compositional data—A review. Anal. Chim. Acta.

[27] Cuadros Rodríguez L., Ruiz Samblás C., Valverde Som L., Pérez Castaño E., González Casado A. (2016). Chromatographic fingerprinting: An innovative approach for food ‘identitation’ and food authentication—A tutorial. Anal. Chim. Acta.

[28] Bagur-González M.G., Pérez-Castaño E., Sánchez-Viñas M., Gázquez-Evangelista D. (2015). Using the liquid-chromatographic-fingerprint of sterols fraction to discriminate virgin olive from other edible oils. J. Chromatogr..

[29] Jiménez Carvelo A.M., Pérez Castaño E., González Casado A., Cuadros Rodríguez L. (2017). One input-class and two input-class classifications for differentiating olive oil from other edible vegetable oils by use of the normal-phase liquid chromatography fingerprint of the methyl-transesterified fraction. Food Chem..

